# Distinguishing Kawasaki Disease from Febrile Infectious Disease Using Gene Pair Signatures

**DOI:** 10.1155/2020/6539398

**Published:** 2020-04-26

**Authors:** Jiayong Zhong, Qingsheng Huang, Yanfei Wang, Huan Gao, Hongling Jia, Jun Fan, Huiying Liang

**Affiliations:** ^1^Institute of Pediatrics, Guangzhou Women and Children's Medical Center, Guangzhou Medical University, No. 9 Jinsui Road, Guangzhou, 510623 Guangdong, China; ^2^Department of Pediatric Cardiology, Guangzhou Women and Children's Medical Center, Guangzhou Medical University, Guangzhou, 510623 Guangdong, China; ^3^Department of Medical Biochemistry and Molecular Biology, School of Medicine, Jinan University, Guangzhou, 510632 Guangdong, China

## Abstract

Kawasaki disease (KD) is an acute systemic vasculitis of childhood with prolonged fever, and the diagnosis of KD is mainly based on clinical criteria, which is prone to misdiagnosis with other febrile infectious (FI) diseases. Currently, there remain no effective molecular markers for KD diagnosis. In this study, we aimed to use a relative-expression-based method k-TSP and resampling framework to identify robust gene pair signatures to distinguish KD from bacterial and virus febrile infectious diseases. Our study pool consisted of 808 childhood patients from several studies and assigned to three groups, namely, the discovery set (*n* = 224), validation set-1 (*n* = 197), and validation set-2 (*n* = 387). We had identified 60 biologically relevant gene pairs and developed a top-ranked gene pair classifier (TRGP) using the first seven signatures, with the area under the receiver-operating characteristic curves (AUROC) of 0.947 (95% CI, 0.918-0.976), a sensitivity of 0.936 (95% CI, 0.872-0.987), and a specificity of 0.774 (95% CI, 0.705-0.836) in the discovery set. In the validation set-1, the TRGP classifier distinguished KD from FI with AUROC of 0.955 (95% CI, 0.919-0.991), a sensitivity of 0.959 (95% CI, 0.925-0.986), and a specificity of 0.863 (95% CI, 0.764-0.961). In the validation set-2, the predictive performance of classification was with an AUROC of 0.796 (95% CI, 0.747-0.845), a sensitivity of 0.797 (95% CI, 0.720-0.864), and a specificity of 0.661 (95% CI, 0.606-0.717). Our study reveals that gene pair signatures are robust across diverse studies and can be utilized as objective biomarkers to distinguish KD from FI, helping to develop a fast, simple, and effective molecular approach to improve the diagnosis of KD.

## 1. Introduction

Kawasaki disease (KD) is an acute childhood self-limited vasculitis syndrome of unknown cause, affecting children younger than five years old [1, 2]. KD affects small and medium blood vessels, especially coronary arteries. The most common sequelae or complications of KD patients are coronary artery lesions, including coronary artery dilation (CAD) and coronary artery aneurysm (CAA), and CAA presents in about 25% of untreated children. KD may be a leading cause of childhood cardiac morbidity in developed countries [2, 3].

The diagnosis of KD mainly relies on clinical features, including fever, extremity change, rash, conjunctivitis, oral changes, and cervical lymphadenopathy [2], which lack specificity. Despite the current development of guidelines, combined with clinical features, echocardiography, and laboratory findings for KD diagnosis, KD patients are still easily misdiagnosed as having febrile infectious diseases such as bacterial infections and viral infections [4]. Thus, there is an urgent need for laboratory markers that can assist KD diagnosis.

The identification of biomarkers in recent studies helps to establish a gold standard test for KD diagnosis. The genome-wide association analysis (GWAS) studies have identified susceptibility genes and loci for KD, including FCGR2A, CASP3, HLA class II, BLK, IPTKC, and CD40 genes [2, 5]. However, none of these genetic signatures associated with KD can be used as diagnosis biomarkers, because of the low disease susceptibility [3]. Serum proteins like N-terminal probrain natriuretic peptide (NT-proBNP) can be used as useful biomarkers for acute and atypical clinical characteristics of KD [6, 7]. Apolipoprotein family, haptoglobin, and fibrinogen-related plasma proteins are found to be high-risk factors and may serve as potential markers of KD [8, 9]. However, these proteins still lack sufficient specificity as KD diagnostic biomarkers. Studies on the role that gene methylation and microRNAs play in KD were active in the past ten years. A recent study found that the methylation level of the HAMP promoter significantly decreases in KD, and this hypomethylation feature can serve as biomarkers for KD diagnosis [10]. Our previous studies found that serum exosomal microRNAs could serve as candidate biomarkers in KD [11] and identified microRNAs that are closely related to coronary aneurysms in KD patients [12]. Although methylation and miRNA are sufficiently specific as KD-related diagnostic biomarkers, they may have limited power due to the small sample size of these studies.

Global gene expression profiles provide novel insights about the disease mechanism and possible diagnostic markers of KD [4, 13–17]. Previous studies used gene expression features to distinguish KD from other febrile control diseases [4, 14, 15]. With gene expression profiles from 162 patients, a study identified features to distinguish KD from adenovirus and GAS infections [14], which are the two most common diseases in KD misdiagnosis. Recently, another large cohort study, involving more than 600 patients, identified differential expression gene (DEG) signatures and used a score-based classifier to distinguish KD from other common mimicking febrile control diseases [15] and got high prediction performance with AUC of 0.96, a sensitivity of 0.82, and specificity of 0.93. These studies analyzed the blood transcriptional profiles and identified the KD biosignatures, which further expanded the scope of KD diagnostic markers. However, these classification methods are developed specifically for the platforms, cohort compositions, and data normalization methods, and are difficult to verify with other public cohort data. Further, the DEG signatures cannot be directly evaluated and verified by qRT-PCR methods, impeding the clinical applications.

The top-scoring pair (TSP) algorithm, which converts a gene expression quantitative value into a binary value of relative expression trends of two genes, provides robust gene pair features without data normalization and has better compatibility in different cohort data [18]. Based on the TSP method, Tan et al. proposed a classifier called the k-Top Scoring Pairs (k-TSP) that uses multiple gene pairs to vote and obtained more accurate results [19]. Afsari et al. had encapsulated the k-TSP method into a convenient R package switchBox [20]. The TSP/k-TSP methods can effectively cope with the data batch effects of different experimental protocols, platforms, and measurement methods [21]. In addition, gene pair features are the relative expression trend between two genes, which can be easily measured and evaluated by the qRT-PCR method. Because of all these advantages, the TSP/k-TSP-based methods have been adopted in several studies [22–25].

In this study, we integrate transcriptional profiling data from several cohorts of childhood KD and febrile infectious (FI) disease including defined bacterial (DB) infection and defined viral (DV) infection and use the k-TSP method and propose a simple resampling-based framework method to identify gene pair signatures to distinguish KD from FI. The new method is robust across diverse studies and should be promising in clinical application.

## 2. Materials and Methods

### 2.1. Data and Preprocessing

In this study, we collected gene expression data related to childhood patients with KD and FI including DB and DV patients from the Gene Expression Omnibus (GEO) database. To identify the robust gene pair signatures, we divided the KD and FI patients into three groups according to the cohort studies: the discovery set (*n* = 224, KD = 78, DB = 52, DV = 94) that consisted of GSE73461 [15]; the validation set-1 (*n* = 197, KD = 146, DB = 23, DV = 28) that consisted of GSE73462 [15] and GSE73463 [15]; and the validation set-2 (*n* = 387, KD = 118, DB = 64, DV = 205) that consisted of GSE48498 [16], GSE16797 [17], GSE68004 [14], GSE40396 [26], GSE38900 [27], and GSE22098 [28].  Table 1 and Supplementary Table 4 show the detailed information for all data sets.

The inclusion criteria of this study included (1) KD, viral infection, and bacterial infection childhood patients; (2) patients in the acute phase of KD or infection and not treated; and (3) patients with whole blood expression profile data, which contain the expression intensity of each gene. In order to eliminate the occurrence of duplicate data, we had checked the raw data using the same chip platform. We have collected 808 samples from 9 data sets that met the above criteria, including 342 cases of KD, 139 cases of DB, and 327 cases of DV (see Table 1 and Supplementary Table 4 for details).

For the data accumulated from the Affymetrix platform GPL570, we downloaded their original chip data format (.cel file) and processed them using the R package affy (version 1.58.0) [29]. For data accumulated from the Illumina platforms GPL10558, GPL6947, and GPL6884, we downloaded the raw data and processed them using the R package limma (version 3.38.3) [30]. In gene annotation, unannotated probesets, as well as probesets that are mapped to multiple genes, were filtered. If several probesets were mapped to the same gene, the highest mean intensity probeset was kept in the data and represented the gene expression value. A total of 16004 common genes across 9 data sets were extracted for subsequent analysis.

### 2.2. Gene Pair Signature Identification

We used the TSP/k-TSP method [18, 19] to transform the gene expression value into the binary values of the gene pair (if the expression of Gene i > Gene j, the value is 1, else 0). A gene pair-based method has the advantage of reducing platform bias and potential batch effects and is robust to any data processing that preserves the gene order [21, 31, 32].

**Figure 1:**
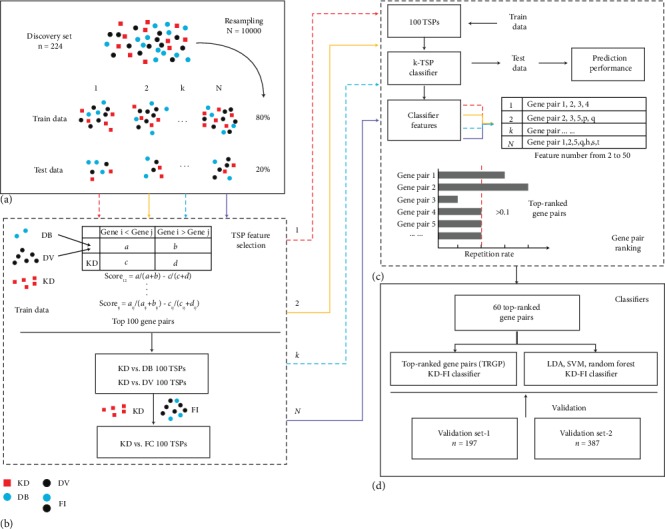
The workflow of the gene pair identification and evaluation for the KD and FI prediction. The workflow has four major analysis steps. (a) Resampling sample space of the discovery set by 10,000 times. Each resampled discovery set is divided into the train data and the test data in a ratio of 8 : 2. (b) Identification of top score gene pairs. (c) Ranking the repeats of the gene pairs form 10,000 k-TSP classifiers. (d) The prediction classifier of KD and FI by the top-ranked gene pairs and validation.

In the discovery set (*n* = 224), we randomly divided 80% of the samples as train data and 20% of the samples as test data for 10,000 times (Figure 1(a)). In each run, we selected the top 100 score gene pairs from KD vs. DB and KD vs. DV in the train data (Figure 1(b)), using Wilcoxon rank-sum method as the filtering function in the switchBox package (version 1.12.0) [20], which is for k-TSP development. Then, we used the combined 200 gene pairs as restricted pairs to reselect 100 top score gene pairs form KD vs. FI in the train data (Figure 1(c)). A k-TSP classifier of KD vs. FI was built from the train data using the 100 top score pairs as features and was evaluated in the test data. If a gene pair is repeated multiple times in the 10,000 k-TSP classifiers, then it has a better classification performance. We collected the gene pairs from the 10,000 runs and selected 60 gene pairs with repeat probability of more than 0.1 as the top-ranked gene pairs (Figures 1(c) and 1(d)).

### 2.3. Random Gene Pair Feature Testing

To test whether the gene pairs with prediction value could be generated by random chances alone, we implemented a permutation test in which the gene symbols and gene expression values were randomly shuffled. We generated 10,000 random discovery sets, in which we identified the top score gene pairs and established k-TSP classifiers as above. We then compared the scores of the gene pair signatures and prediction performance of classifiers from the random discovery set and discovery set.

### 2.4. Top-Ranked Gene Pair (TRGP) Classifier

We used the top-ranked gene pair signatures to develop a simple voting classifier called the top-ranked gene pair (TRGP) classifier. If the gene pair *p*_ij_ is with the expression Gene i > Gene j (Equation (1)), it votes for KD and assigns a value of 1; otherwise, it votes for FI and assigns a value of -1.

(1) $$\begin{array}{c} p_{\text{ij}}=\left\{\begin{array}{c} 1\;\left(\text{Gene i}>\text{Gene j}\right),\\ -1\;\left(\text{Gene i}<\text{Gene j}\right). \end{array}\right. \end{array}$$

*N* is the number of gene pair signatures. The classification score is equal to the average scores of all the gene pairs (Equation (2)), which is a judgment indicator of the classifier.

(2) $$\begin{array}{c} \text{Classification score}=\frac{\sum p_{\text{ij}}}{N}. \end{array}$$

The higher the score, the higher probability of KD classification. To determine the optimal number of gene pairs for the classifier, we used from one to sixty gene pair signatures to develop the TRGP classifiers. The balanced accuracy and AUROC on the discovery set were used to evaluate the performance of the TRGP classifier with different number of gene pairs.

### 2.5. Linear Discriminant Analysis (LDA), Support Vector Machines (SVM), and Random Forest Classifier

The 60 top-ranked gene pair signatures were used to construct LDA, SVM, and random forest classifiers. Tenfold cross-validation was used to determine the most critical gene pair features for each classifier that best distinguish KD from FI patients in the discovery set. The linear SVM function in caret (version 6.0.84) package [33] was used to identify essential gene pairs, and then the SVM classifier was built by the e1071 (version 1.7.1) package [34]. The Boruta (version) R package (version 6.0.0) [35] was used to identify essential gene pairs, and the random forest classifier was built by the randomForest (version 4.6.14) R package [36].

### 2.6. Performance Evaluation

We evaluate the performance including sensitivity, specificity, accuracy, and AUROC of the TRGP, LDA, SVM, and random forest classifiers in the discovery set, validation set-1, and validation set-2. The sensitivity is defined as the proportion of correctly predicted KD in all actual KD patients, and the specificity is defined as the proportion of the FI in all actual FI patients. The accuracy is defined as the proportion of correctly identified patients of all KD and FI. The balanced accuracy is equal to the mean of KD prediction accuracy and FI prediction accuracy.

We used the pROC package (version 1.14.0) [37] to analyze the top-ranked gene pair classifier and calculated the AUROC in three data sets.

### 2.7. Biological Effects of the Top-Ranked Gene Pairs

To roughly assess the classification effect of the top-ranked gene pairs, we performed the t-Distributed Stochastic Neighbor Embedding (t-SNE) clustering using the Rtsne (version 0.15) R package [38], setting a perplexity to 60 and a theta to 0.5.

To study the correlation between the 60 top-ranked gene pairs and the differential expressed genes (DEGs), we used the limma package (version 3.38.3) [30] to identify DEGs from KD and FI in the discovery set. DEGs of KD vs. DV and KD vs. DB were also calculated. Then, we used the red triangle and green square symbols to represent the top-ranked gene pairs and draw the volcano map of the log scale fold change and detection of the *P* value.

The Clusterprofiler (version 3.8.1) R package [39] was used for KEGG enrichment analysis for the Gene i from the left side and Gene j from the right side of 60 top-ranked gene pairs.

## 3. Results

### 3.1. Identification of Diagnostic Gene Pairs

**Figure 2:**
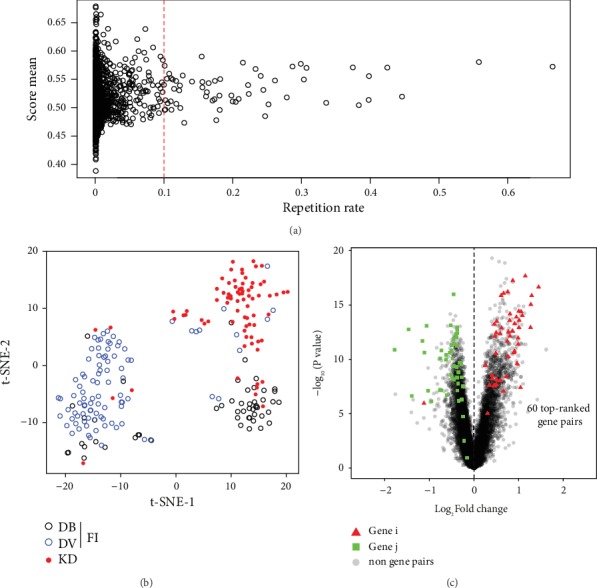
The 60 top-ranked gene pairs. (a) The repetition rate and mean scores of gene pairs in 10,000 resampled train data. Each point represents a gene pair, the red line is the threshold line with a repetition rate of 0.1, and we take a repetition rate greater than 0.1 as the top-ranked gene pairs. (b) Unsupervised t-SNE classification of the discovery set was performed using 60 top-ranked gene pairs. (c) The overlap of differentially expressed genes (KD vs. FI in the discovery set) with 60 top-ranked gene pairs, and Gene i (red triangle) and Gene j (green square) represent the genes to the left and the right of gene pairs, respectively.

We collected 78 KD and 146 FI including 94 DV and 52 DB childhood patients from GSE73461 [15] (Table 1) as the discovery set, from which 2522 top score gene pairs had been identified (Supplementary Table 1) according to the workflow described in Figure 1 and Materials and Methods. Most of the gene pairs have a repetition rate less than 0.01 in the 10,000 k-TSP classifiers generated by the resampling runs, while 60 gene pairs have a high repetition rate more than 0.1 and the average scores of these gene pairs are about 0.53 (Figure 2(a), Supplementary Table 1). These 60 high repetition gene pairs are marked as top-ranked gene pairs and have provided support to the efficient clustering of KD and FI in the t-SNE plot (Figure 2(b)).

Besides, we found that these top score gene pairs were not generated by random chances. The score of gene pairs from random discovery data of 0.35 (95% CI, 0.25-0.42) is significantly smaller than the score of 0.57 (95% CI, 0.41-0.70) from nonrandom discovery set (*P* < 0.0001, two-sample Kolmogorov-Smirnov test) (Supplementary Figure S2A). Gene pairs with higher scores could have better prediction performance [18, 21]. The number of features of the k-TSP classifier developed in the random train data is also larger than that in the nonrandom train data (Supplementary Figure S2B). Further, the result shows that the AUROC of the k-TSP classifier in the random train data and test data reduced from 0.98 to 0.48, while on the discovery set, the AUROC of the k-TSP classifier in the train and test data was reduced from 0.95 to 0.89. These results indicated that the top score gene pairs identified from the nonrandom data are more robust and yield significantly more predictive information than in random chances.

### 3.2. Evaluation of the Biological Effect of Top-Ranked Gene Pairs

Next, we investigated whether the gene pair signatures are biologically relevant. We analyzed the overlap of DEGs and genes from top-ranked gene pairs (Gene i > Gene j) in the discovery set and found that nearly all of the Gene i were upregulated genes, and the Gene j were downregulated genes (Figure 2(c)) in KD vs. FI. The same trend is found in the comparison of KD vs. DV and KD vs. DB (Supplementary Figure S4). These results suggested that the identified top-ranked gene pairs are biologically related to the DEGs from KD and control diseases.

In addition, functional analysis of the Gene i in 60 top-ranked gene pairs, which upregulated in KD, shows enrichment of TNF signaling pathway, PI3K-Akt signaling pathway, and NOD-like receptor signaling pathway (Supplementary Figure S5A). While functional analysis of the Gene j, which downregulated in KD and upregulated to FI, shows enrichment of infection-related pathways such as measles, influenza A, and Epstein-Barr virus infection pathways (Supplementary Figure S5B). These results reveal that the gene pairs we have identified are biologically related to the functional pathway.

### 3.3. Evaluation and Validation of the Gene Pair Signatures in TRGP Classifier

To obtain the best prediction performance using the 60 top-ranked gene pair features, we constructed TRGP, LDA, SVM, and random forest classifiers (see Materials and Methods). Then, we evaluated the predictive value of the top-ranked gene pairs and classifiers in two independent validation data sets, including the validation set-1 (GSE73462 and GSE73463) and validation set-2 (GSE40396, GSE48498, GSE16797, GSE38900, and GSE22098).

**Figure 3:**
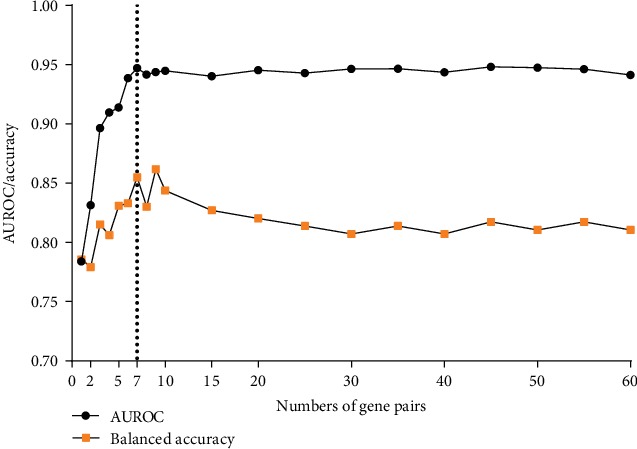
The AUROC and balanced accuracy of number features in a k-TSP classifier selected from the 60 top-ranked gene pairs in the discovery set. We selected gene pairs from 1 to 60 to develop the TRGP classifier and found that the TRGP classifier with seven of the top gene pairs (dotted line) optimally achieved the best AUROC and balanced accuracy performance.

According to the process described in Materials and Methods, when we selected the first 7 gene pairs (Table 2) from the 60 top-ranked gene pairs as diagnostic features, the TRGP classifier achieved the best performance in the discovery set, with the AUROC of 0.95 and the balanced accuracy of 0.86 (Figure 3). Increasing the number of gene pairs to the classifier did not improve the prediction performance but slightly reduced balance accuracy (Figure 3).

**Figure 4:**
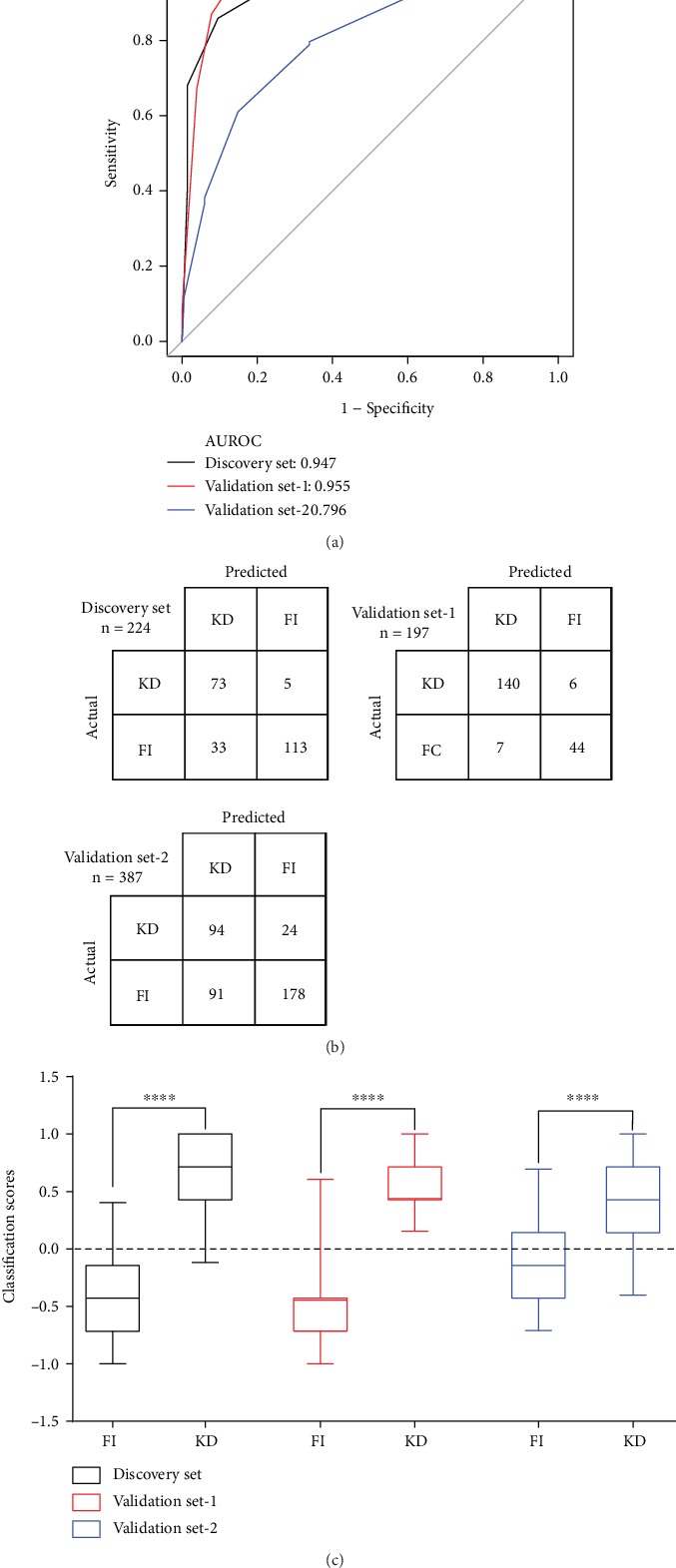
Prediction performance of the TRGP classifier using the seven top-ranked gene pairs. (a) The ROC curve, (b) the prediction confusion matrix, and (c) the classification scores of FI and KD patients in the discovery set (black), validation set-1 (red), and external validation set-2 (blue). In box plots (c), the horizontal lines, box edges, and whiskers represent the median, interquartile ranges, and 95% percentile range, respectively. The dotted line represents the threshold of classification, and the classification scores > 0 are predicted to be KD, otherwise FI. AUROC: area under the receiver-operating characteristic curve. A two-tailed unpaired Student's *t*-test was used for statistical comparison of classification scores between FI and KD patients. ^∗^*P* < 0.05,^∗∗^*P* < 0.01,^∗∗∗^*P* < 0.001, and^∗∗∗∗^*P* < 0.0001.

Based on the voting rules of selected gene pairs, the TPGP classifier calculates a classification score for each case, and 0 is the boundary threshold for KD and FI. The larger score of the subject, the greater the probability of KD, otherwise of FI. There were significant differences in the classification scores of KD and FI patients both in the discovery set (*P* < 0.0001, two-tailed unpaired Student's *t*-test) and two of the validation sets (*P* < 0.0001 for validation set-1 and *P* < 0.0001 for validation set-2; two-tailed unpaired Student's *t*-test) (Figure 4(c)). The classification scores of KD patients were significantly higher than those of the FI patients, including DV and DB patients (Supplementary Figure S3), in all data sets.

We then tested the prediction performance of TRGP classifier by calculating the AUROC, sensitivity, and specificity for each dataset separately. In the discovery set, the prediction AUROC was 0.947 (95% CI, 0.918-0.976), with sensitivity of 0.936 (95% CI, 0.872-0.987) and specificity of 0.774 (95% CI, 0.705-0.836) (Figure 4(a), Table 2). Because of the higher proportion of the KD case, the predictive performance was better in the validation set-1, with the AUC of 0.955 (95% CI, 0.919-0.991), sensitivity of 0.959 (95% CI, 0.925-0.986), and specificity of 0.863 (95% CI, 0.764-0.961). In the independent validation set-2, the performance was reduced with the AUROC of 0.796 (95% CI, 0.747-0.845), a sensitivity of 0.797 (95% CI, 0.720-0.864), and a specificity of 0.661 (95% CI, 0.606-0.717). This performance reduction may be due to the higher heterogeneity of the validation set-2, including differences in KD diagnosis and differences of infectious pathogen composition in DV/DB cases. Nonetheless, with the TRGP classifier, we correctly distinguished 79% KD patients and 66.1% FI patients in the validation set-2 (Figure 4(b)).

In clinical practices, the incomplete KD (inKD) is more difficult to diagnose than the complete KD (cKD) [2]. We noticed that in the GSE68004 dataset (validation set-2), there were no significant differences in the classification scores of cKD and inKD patients (Supplementary Figure S7A). We correctly distinguished 73.7% of cKD and 76.9% of inKD subjects (Supplementary Figure S7B), showing that the TRGP classifier has a similar predictive performance on cKD and inKD.

Taken together, these results reveal that the gene pair signatures and the TPGP classifier can effectively distinguish KD and FI in the discovery set and independent validation sets.

### 3.4. Evaluation and Validation of the Gene Pair Signatures in Other Classifiers

To compare the performance of other weighted linear classifier and nonlinear classifiers, we then applied the 60 top-ranked gene pairs to the LDA, SVM, and random forest classifiers and compared the performance of these classifiers. The number of gene pairs was determined by a 10-fold cross-validation in the discovery set. According to the cross-validation, the best LDA classifier used all 60 gene pairs, while the best SVM used 12 gene pairs (Supplementary Table 2) and the best random forest classifier used 56 gene pairs (Supplementary Figure S6).

The AUROC was used to evaluate the predictive performance of these classifiers. The best performance for discrimination of KD and FI in the discovery set was the random forest (1.00), followed by LDA (0.980), SVM (0.956), and TRGP (0.947) (Table 3). In the two validation sets, TRGP achieved the best performance (with AUROC of 0.955, 0.796), followed by the random forest (0.828, 0.751), SVM (0.791, 0.671), and LDA (0.860, 0.601). The performance differences in the discovery set were small, but the TRGP performed the best overall in the validation data set and used the least number of gene pairs. These results reveal that the gene pair signatures can be flexibly applied to different classifiers and achieve similar prediction performance. Notably, the TRGP classifier uses the simplest decision rules and the fewest number of features to achieve a reliable predictive performance.

## 4. Discussion

The current guidelines [2, 40] for KD diagnosis only rely on clinical signs, but there are similar clinical signs between Kawasaki disease and febrile control diseases, which can easily lead to either delay of diagnosis for KD or overtreatment with IVIG for actual benign febrile disease patients [41]. Thus, it is necessary to find laboratory testing markers to improve the diagnosis of KD. In this study, based on the relative gene expression analysis method, we integrated the public data of whole blood gene expression profiles from 9 data sets, then identified 60 gene pair signatures related to classification of KD and FI, and finally developed a seven top-ranked gene pair voting classifier TRGP to accurately distinguish KD from FI.

High-throughput transcriptome profiles provide a tremendous amount of information and enable us to collect molecular markers, discriminate disease subtypes, predict clinical outcomes, and reveal specific changes to disease progression. Gene expression data have been used in multiple studies to distinguish febrile children with bacterial or viral infection [27, 42]. Popper et al. used 38 gene expression features to distinguish KD patients from adenovirus infection with an overall accuracy of 0.903 in 41 subjects. Subsequently, Jaggi et al. developed 25 and 10 gene signature KNN classifiers to differentiate KD and adenovirus infection with a sensitivity of 0.92 in the validation dataset and to differentiate KD and GAS bacterial infection with a sensitivity of 0.87, respectively [14]. In a recent study, Wright et al. used the whole blood expression profiles to identify 13 gene features and successfully distinguish acute KD and febrile infectious/inflammatory disease in a large cohort of 606 patients with a sensitivity of 0.859, a specificity of 0.891, and AUROC of 0.946 in validation [15]. These studies are very attractive, demonstrating that we can extract molecular markers of diagnostic value for KD from transcriptome profiles.

The methods applied in previous studies identified genetic features whose differential expression is highly correlated with disease subtypes, such as genes that may be over- or underexpressed in KD relative to febrile control disease. In this case, a gene whose expression level is up- or downregulated above some certain threshold is considered to be a candidate marker of the disease. However, many factors, such as study design and the data normalization methods, largely influence the statistically significant changes of features in gene expression analysis. Unfortunately, the identified molecular features are rarely reproducible or even without overlap in different platforms or in different clinical studies. Thus, the biggest challenge in identifying useful molecular markers is to develop robust and accurate classifiers for multiple platforms and studies. To overcome this challenge, the relative expression analysis framework TSP/k-TSP has been proposed [18, 19], which is characterized by replacing the expression levels of all the genes with the relative rankings of expression values, evaluating the change of relative order among two genes from one phenotype to another.

In this study, based on the k-TSP method, we applied a resampling framework to identify robust gene pair features and distinguish Kawasaki disease from febrile control disease. The k-TSP methods are parameter-free methods that only rely on the relative ordering of gene expression values and are robust to all normalization methods [19]. As the data normalization is not needed, these methods have therefore proven to be useful in the integration of data across different studies and platforms to increase sample size and to avoid spurious discovery [43]. Ao et al. used the in-sample relative expression ordering (REO) method, developed based on the principle of relative expression analysis to integrate the expression profile data related to cirrhosis and hepatocellular carcinoma from the GEO and TCGA databases and identified robust molecular signatures for the early diagnosis of HCC [24]. Sandhu et al. developed a robust Pancreatic Cancer Overall Survival Predictor (PCOSP) model based on the k-TSP method, integrating multiple datasets including sequencing and chip array profiles, and achieved better predictive performance than previous models [25].

The discovery set and validation set-1 of this study are derived from Wright's study [15], compared with which, the seven top-ranked gene pair classifier achieved a similar prediction performance, with a sensitivity of 0.959, a specificity of 0.863, and AUROC of 0.955 in the same validation set-1 (Table 3). Taking advantage of the TSP/k-TSP methods, we integrated other six whole blood transcriptome datasets as an independent external validation dataset (validation set-2, Table 1), in which the prediction performance of the identified gene pairs were reduced, with AUROC of 0.796, a sensitivity of 0.797, and a specificity of 0.662.

The decline performance may be related to the heterogeneity between discovery set and the validation set-2.We noticed that there were differences in the age of KD and FI patients among the three data sets (Supplementary Table 4). In the discovery and validation set-1, the median age of KD was 27 and 34 months [15], respectively. In validation set-2, the KD patients came from 3 studies and the weighted median age was 40.7 months [14, 16, 17], which was larger than in the discovery and validation set-1. And the age of FI in validation set-2 also varied quite a bit compared to the discovery and validation set-1 (Supplementary Table 4). Previous studies have shown that the regulation expression of many functional genes was in an age-dependent fashion [44, 45]. And the incidence rates and outcome of coronary artery aneurysms for Kawasaki disease are age-related [46]; thus, the expression pattern associated with KD may be also affected by ages. Therefore, one of the reasons for the prediction performance decline in validation set-2 may be age differences. In addition to age, we have also noticed differences of race/ethnicity in the three data sets. The ethnic proportions of KD in the discovery set and validation set-1 were relatively uniform; the main populations were White, Hispanic, and Asian/other (from 19.4 to 50%) [15], while in validation set-2, the main populations were White (52%) in Jaggi's study [14] and Asian (100%) in Ogihara's [16] and Ogata's [17] studies. Previous studies have shown that there are certain ethnic expression differences in healthy conditions and various diseases such as diabetes, breast cancer, and hypertension [47–49]. The prevalence of KD varies considerably among races, and it is more common among Asians [50], which suggests race/ethnicity-related genetic differences associated with susceptibility variance. Therefore, the race/ethnicity-related genetic differences may also partly explain why the prediction performance reduced in validation set-2. Other differences including diagnosis of KD, missed diagnosis KD in the FI, and differences of bacterial infectious subtypes could affect the prediction performance of the result too. However, prediction of the TRGP classifier yields AUC = 0.795 which is acceptable in clinical practice and may still improve the diagnosis of KD. Despite the above deficiencies, our results are sufficient to demonstrate that the identified gene pairs have robust predictive value in multiple datasets. Compared to previous KD studies, our framework enables the integration of expression profiles from multiple datasets to prove that the identified characteristic gene pairs are not spurious findings.

Notably, the gene pairs identified by the TSP/k-TSP method can be directly validated by using qRT-PCR [23, 51, 52], a more targeted and affordable technology in clinical practice. Zak et al. had identified gene pair signatures of tuberculosis risk from RNA sequencing data and successfully applied qRT-PCR for validation [23], showing the possibility of clinical promotion of this framework approach. Previous studies used chip-detected intensity values as genetic signatures, which usually did not have linear correlations between different platforms and measurement technologies and could not be directly verified by the qRT-PCR method. Therefore, compared with previous studies, the gene pair signatures in this study are more feasible in further validation studies.

The 60 top-ranked gene pair features achieved similar prediction performance by applying multiple classification algorithms (Table 3), indicating that the signatures are associated with differentiation between KD and FI. Besides, our results showed that these gene pairs overlapped with the phenotype-related DEGs (Figure 2(c), Supplementary Figure S4). And functional analysis revealed that the genes in Gene i of the 60 top-ranked gene pairs were associated with the TNF signaling pathway, PI3K-Akt signaling pathway, and NOD-like receptor signaling pathway (Supplementary Figure S5A). TNF-*α* is a pleiotropic inflammatory cytokine in the acute phase of Kawasaki disease and has essential physiological functions in vasculitis of KD [53]. The polymorphism of the NOD1 is associated with a high risk of KD [54], and the NOD-like receptor is involved in the physiological process of acute KD [55]. While the genes in Gene j are associated with infection-related pathways such as measles, influenza A, and Epstein-Barr virus infection pathways (Supplementary Figure S5B), these results indicate that the gene pairs we have identified are not only statistically related to phenotype but also biologically relevant to the phenotype.

We recognize several limitations in this study. The heterogeneity of the multiple datasets may limit the predictive performance of this study, as mentioned above. However, this problem mainly exists in the validation set-2, because in the discovery set and the validation set-1, the KD and FI patients are strictly unified, as described in Wright' study [15]. Second, we lacked detailed clinical information of the subjects and do not know the days of fever in KD patients, but all KD patients in this study belong to the acute phase of fever as the study cohorts described. Finally, in our study, there is still a lack of transcriptome data measured using other techniques, such as RNA-Seq and qRT-PCR. We will recruit subjects to provide this data in future studies to evaluate the KD diagnostic value of gene pair features.

In conclusion, our results demonstrate that gene pairs can be used as robust biomarkers to distinguish between KD and FI. Furthermore, these gene pair signatures are biologically relevant and can be easily tested and validated by the qRT-PCR method. The seven top-ranked gene pair TRGP classifier can also be used as a single sample predictor in the clinical environment. The above results warrant additional investigation; if confirmed further, it could help to establish a rapid and inexpensive diagnostic method to improve the early diagnosis of KD.

## 5. Conclusions

In this study, our results demonstrate that gene pairs can be used as robust biomarkers to distinguish between KD and FI. Furthermore, these gene pair signatures are biologically relevant and can be easily tested and validated by the qRT-PCR method. The seven top-ranked gene pair TRGP classifier can also be used as a single sample predictor in the clinical environment. The above results warrant additional investigation; if confirmed further, it could help to establish a rapid and inexpensive diagnostic method to improve the early diagnosis of KD.

## Figures and Tables

**Table 1:** Description of data sets used in this study.

Data sets	Accession	Platform	Total (*N*)	Sex males (%)	KD (*N*)	DB (*N*)	DV (*N*)	Ref.
Discovery set Validation	GSE73461	GPL10558	224	56.2	78	52	94	Wright et al. [15]

set-1	GSE73462	GPL6947	51	52.9	0	23	28	Wright et al. [15]
	GSE73463	GPL10558	146	59.4	146	0	0	Wright et al. [15]

Validation set-2	GSE48498	GPL570	12	NA	12	0	0	Ogihara et al. [16]
	GSE16797	GPL570	17	NA	17	0	0	Ogata et al. [17]
	GSE68004	GPL10558	125	58.4	89	17	19	Jaggi et al. [14]
	GSE40396	GPL10558	43	58.1	0	8	35	Hu et al. [26]
	GSE38900	GPL6884 GPL10558	151	51.0	0	0	151	Mejias et al. [27]
	GSE22098	GPL6947	39	48.7	0	39	0	Berry et al. [28]

**Table 2:** The 7 top-ranked gene pairs.

Gene pair IDs	Gene i EntrezID	Gene j EntrezID	Gene i symbol	Gene j symbol	Repetition rate
GenePair1	9778	9862	KIAA0232	MED24	0.665
GenePair2	9586	3429	CREB5	IFI27	0.557
GenePair3	10857	7384	PGRMC1	UQCRC1	0.446
GenePair4	7739	5096	ZNF185	PCCB	0.424
GenePair5	83999	8454	KREMEN1	CUL1	0.397
GenePair6	54602	116832	NDFIP2	RPL39L	0.397
GenePair7	64127	593	NOD2	BCKDHA	0.382

**Table 3:** The predictive performance of TRGP, LDA, SVM, and random forest classifier.

Classifier	Data sets	Number of gene pairs	Accuracy	Sensitivity	Specificity	Precision	Balanced accuracy	AUROC
LDA	Discovery set	60	0.978	0.993	0.949	0.973	0.971	0.980
	Validation set-1		0.898	0.882	0.904	0.763	0.893	0.860
	Validation set-2		0.589	0.543	0.695	0.802	0.619	0.601

SVM	Discovery set	12	0.955	0.979	0.910	0.953	0.945	0.956
	Validation set-1		0.817	0.980	0.760	0.588	0.870	0.790
	Validation set-2		0.698	0.699	0.695	0.839	0.697	0.671

Random forest	Discovery set	56	1.000	1.000	1.000	1.000	1.000	1.000
	Validation set-1		0.868	0.961	0.836	0.671	0.898	0.828
	Validation set-2		0.788	0.866	0.610	0.835	0.738	0.751

TRGP	Discovery set	7	0.830	0.936	0.774	0.689	0.855	0.947
	Validation set-1		0.934	0.959	0.863	0.952	0.911	0.955
	Validation set-2		0.703	0.797	0.662	0.508	0.729	0.796

## Data Availability

The public gene expression data used in this study can be accessed on Gene Expression Omnibus (GEO), and the data used to support the findings of this study are included within the article.

## Supplementary Materials

Supplementary 1 Figure S1: repeatability saturation curve of the top 100 gene pairs in resampling data sets. Supplementary Figure S2: comparison of gene pairs with random discovery set. Supplementary Figure S3: the classification scores of DB, DV, and KD patients in the discovery set, validation set-1, and validation set-2. Supplementary Figure S4: the overlap of DEGs with 60 top-ranked gene pairs. Supplementary Figure S5: KEGG pathway enrichment of 60 top-ranked gene pairs. Supplementary Figure S6: the importance of 60 top-ranked gene pair signatures in random forest classifier. Supplementary Figure S7: the prediction performance of TRGP classifier in the cKD and inKD patients.

Supplementary 2 Table 1: the 2522 top scores gene pairs and the 60 top-ranked gene pairs. Supplementary Table 2: the performance of TRGP classifier in different GEO data sets. Supplementary Table 3: the importance of 12 gene pair signatures in the SVM classifier. Supplementary Table 4: the detailed information of each study of the discovery validation.
