# Supplementary material for: Distinguishing Kawasaki Disease from Febrile Infectious Disease Using Gene Pair Signatures
Source: Biomed Res Int. 2020 Apr 26;2020:6539398. doi: 10.1155/2020/6539398 (PMC7201505; doi:10.1155/2020/6539398)
Supplement: Supplementary 1 — Figure S1: repeatability saturation curve of the top 100 gene pairs in resampling data sets. Supplementary Figure S2: comparison of gene pairs with random discovery set. Supplementary Figure S3: the classification scores of DB, DV, and KD patients in the discovery set, validation set-1, and validation set-2. Supplementary Figure S4: the overlap of DEGs with 60 top-ranked gene pairs. Supplementary Figure S5: KEGG pathway enrichment of 60 top-ranked gene pairs. Supplementary Figure S6: the importance of 60 top-ranked gene pair signatures in random forest classifier. Supplementary Figure S7: the prediction performance of TRGP classifier in the cKD and inKD patients. [file 6539398.f1.pdf]

Supplementary Figures

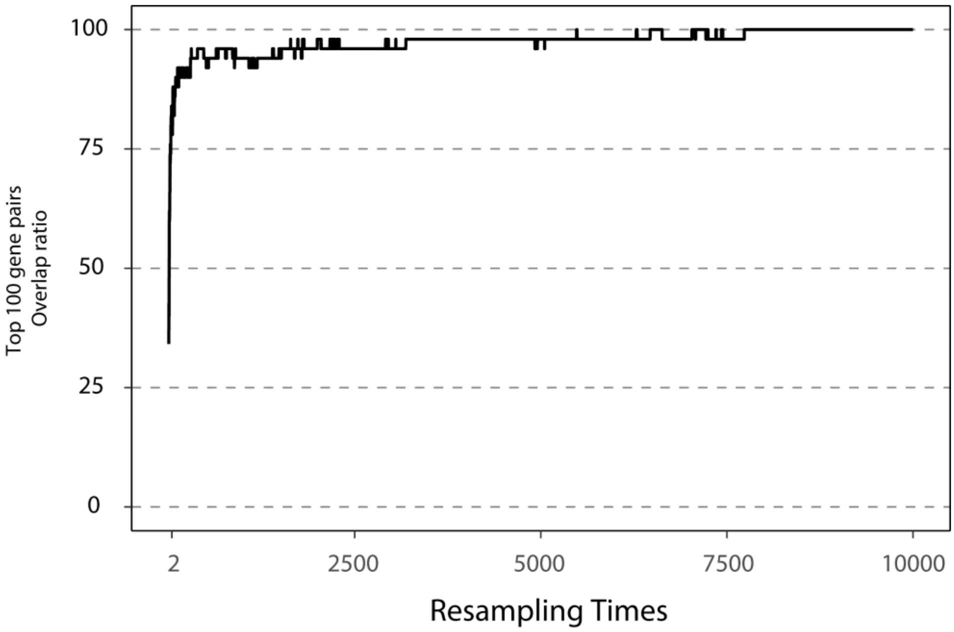

Supplementary Figure S1. Repeatability saturation curve of the top 100 gene pairs in resampling data sets. The top 100 gene pairs identified from 10000 resampling sample spaces as standard, were compared with top 100 gene pairs from 2,3,...,N(N<= 10000) resampling sample spaces, and their intersection ratio were shown in Y-axis.

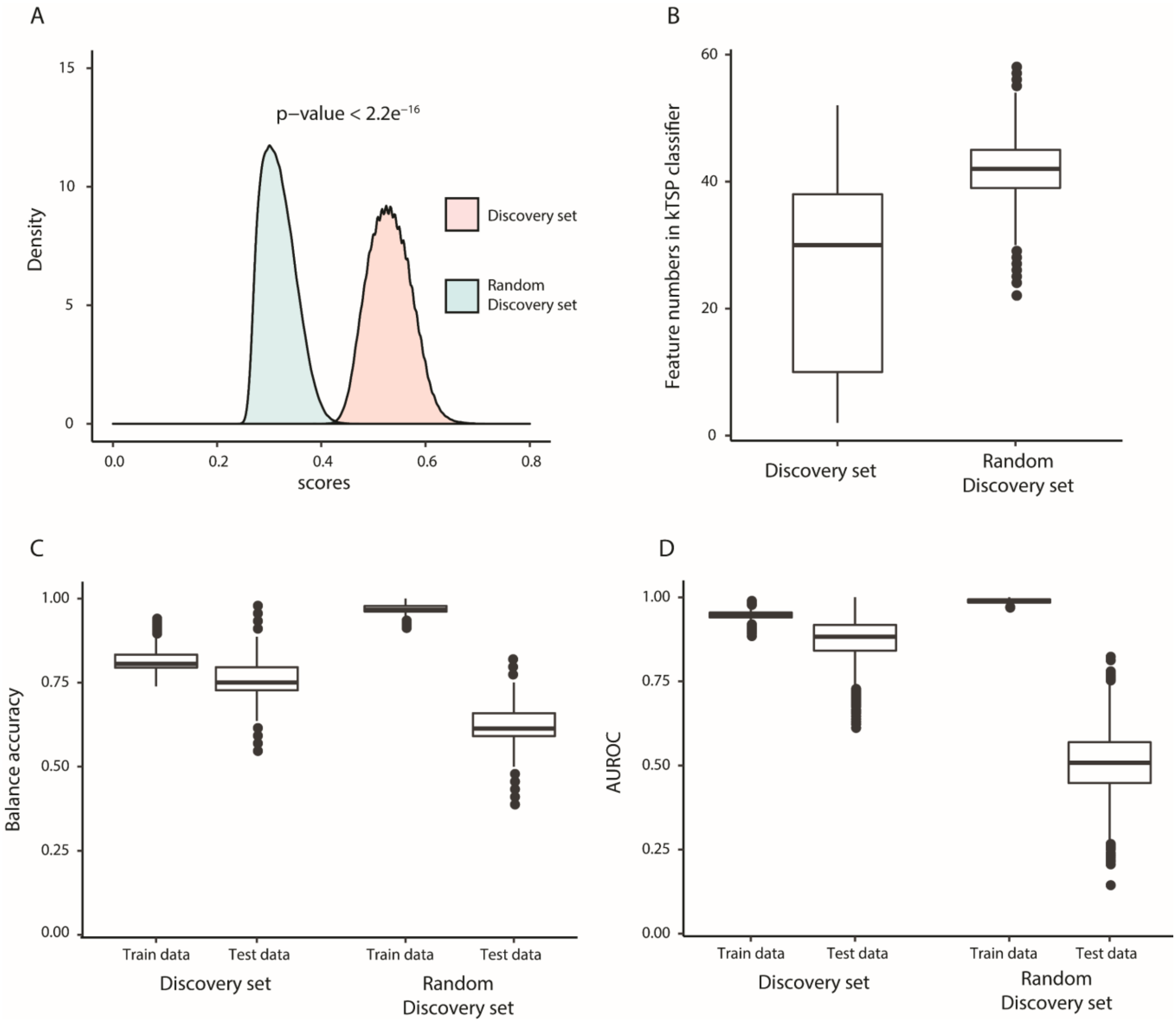

Supplementary Figure S2: Comparison of gene pairs with random discovery set. (A) Density plot showing the score distribution of gene pairs identified form discovery set and discovery set with random reshuffling of gene IDs and gene expression values (random discovery set). P value shows the two-sample Kolmogorov-Smirnov test for the distributions of the scores. (B)The number of gene pairs used in training k-TSPs classifier in the 10000 resampling data sets. (C-D) The AUROC, balanced accuracy predictive performance of k-TSPs classifier in train and test data from discovery set and random discovery set. In box plots, horizontal lines represent the median, the box edges represent the interquartile ranges, whiskers represent the range of 95% percentile range and dots represent the outliers.

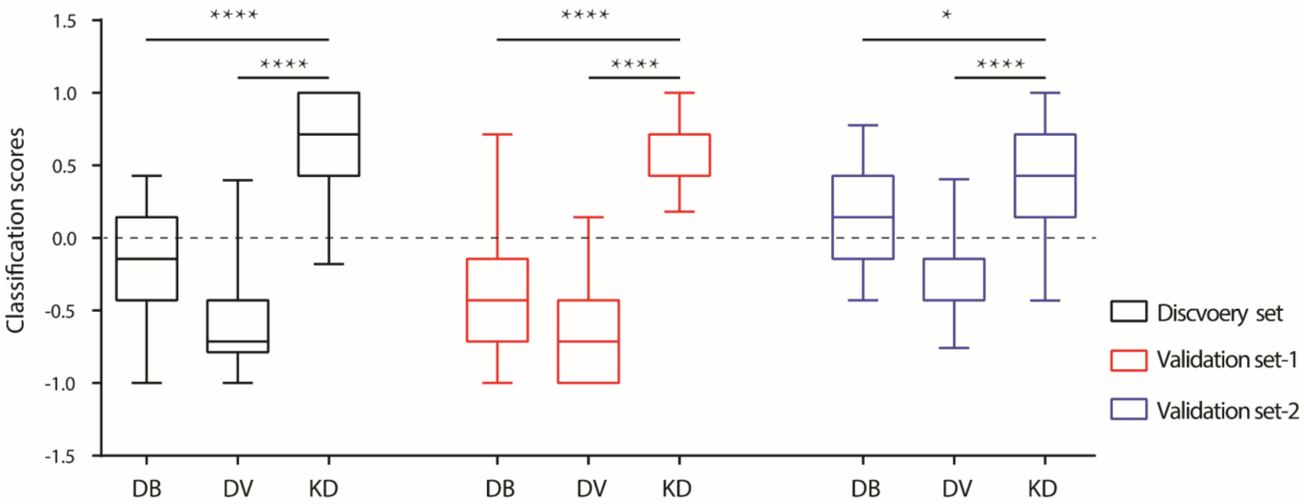

Supplementary Figure S3. The classification scores of DB, DV and KD patients in discovery set, validation set-1 and validation set-2. Two-tailed unpaired Student's t-test was used for statistical comparison of classification scores between DB/DV and KD patients. *P < 0.05.*P < 0.05, **P < 0.01, ***P < 0.001, ****P < 0.0001.

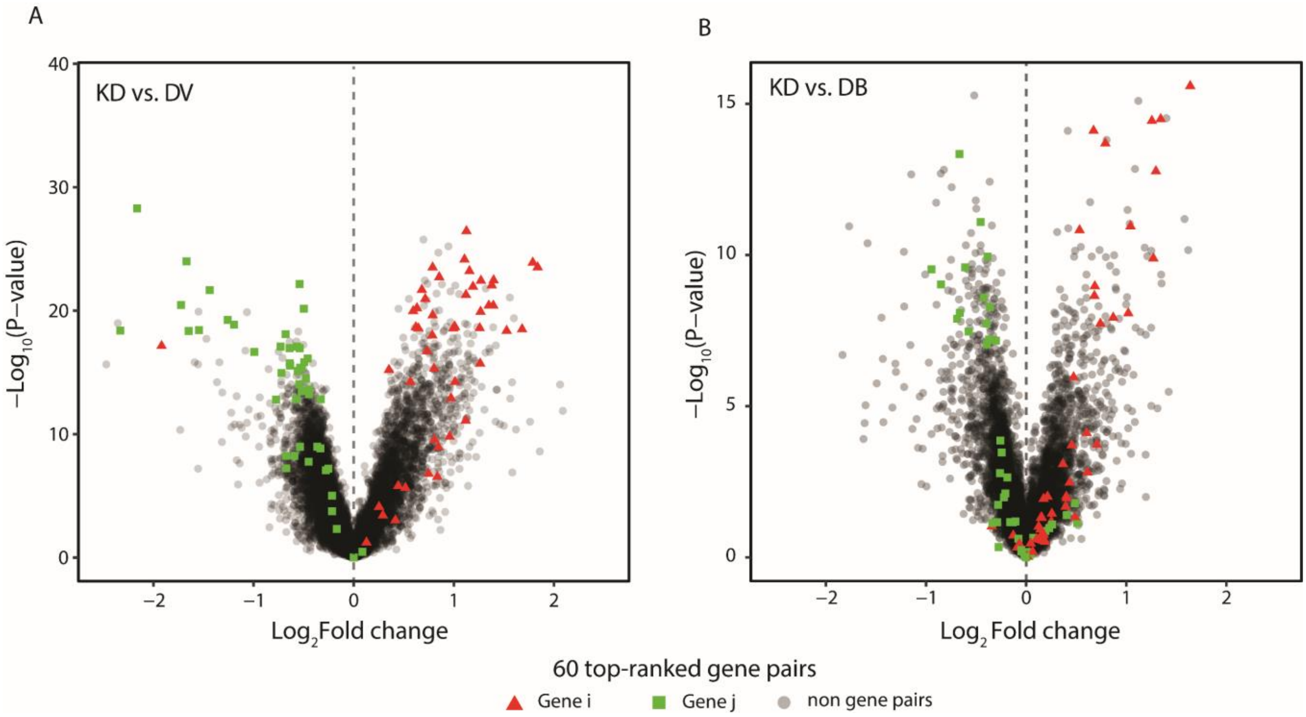

Supplementary Figure S4. The overlap of DEGs with 60 top-ranked gene pairs. The DEGs were identified by KD vs. DV (A) and KD vs. DB (B) in Discovery data set, respectively. Gene i (red triangle) and Gene j (green square) represent the genes to the left and right of gene pairs, respectively.

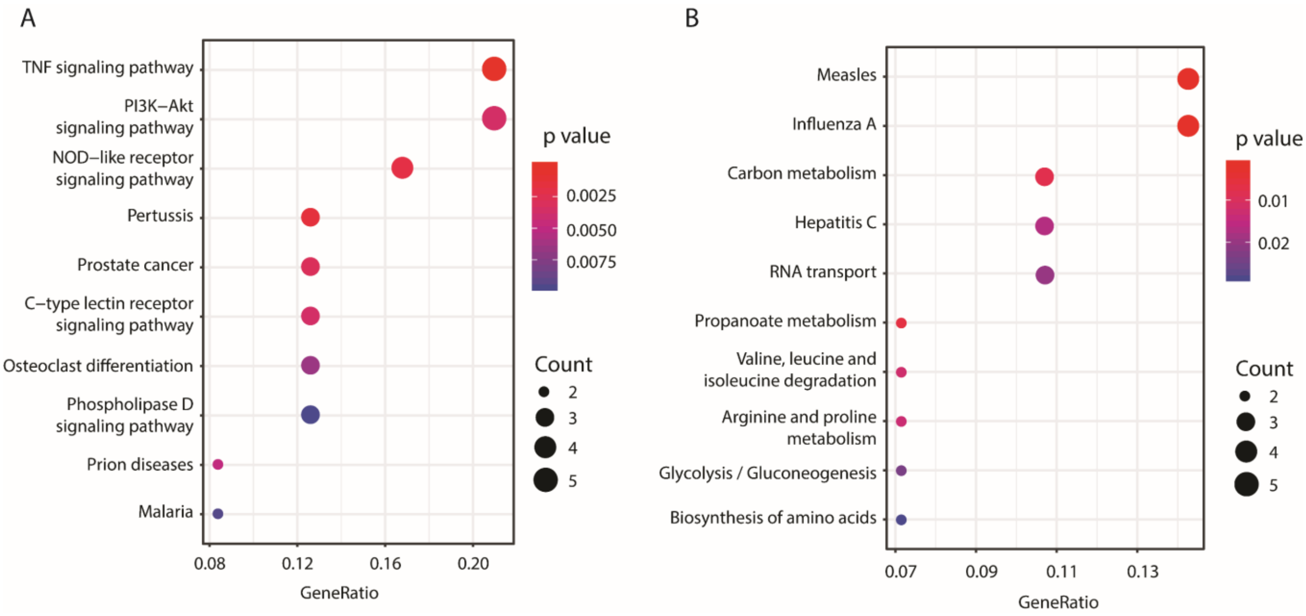

Supplementary Figure S5. KEGG pathway enrichment of 60 top-ranked gene pairs. The top 10 KEGG enrichment pathways of Gene i (A) and Gene j (B).

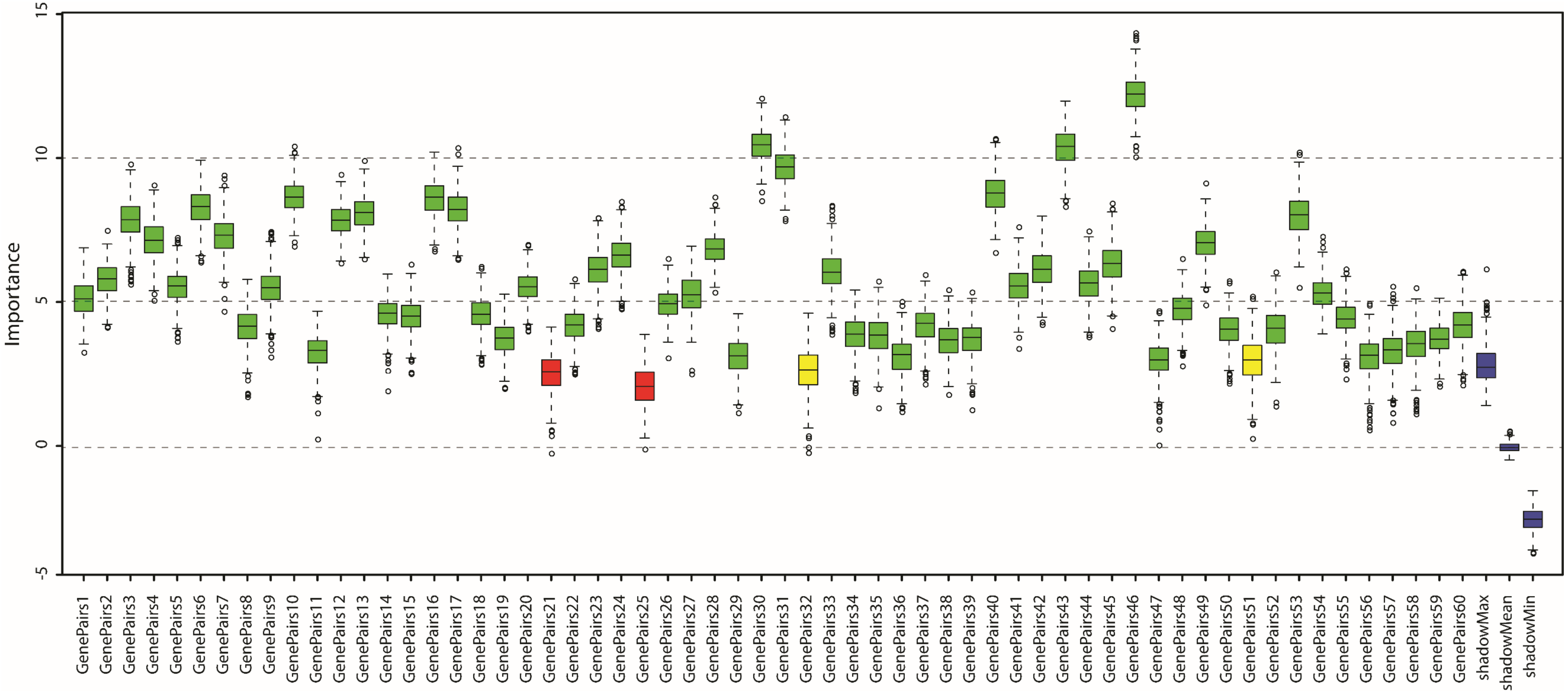

Supplementary Figure S6. The importance of 60 top-ranked gene pair signatures in random forest classifier. Red, yellow, and green indicate that the gene pairs with low, medium and high importance in random forest classifier respectively. Blue indicates the importance of shadow features. Gene pairs with importance higher than the shadow features are considered to have predictive performance in the random forest classifier.

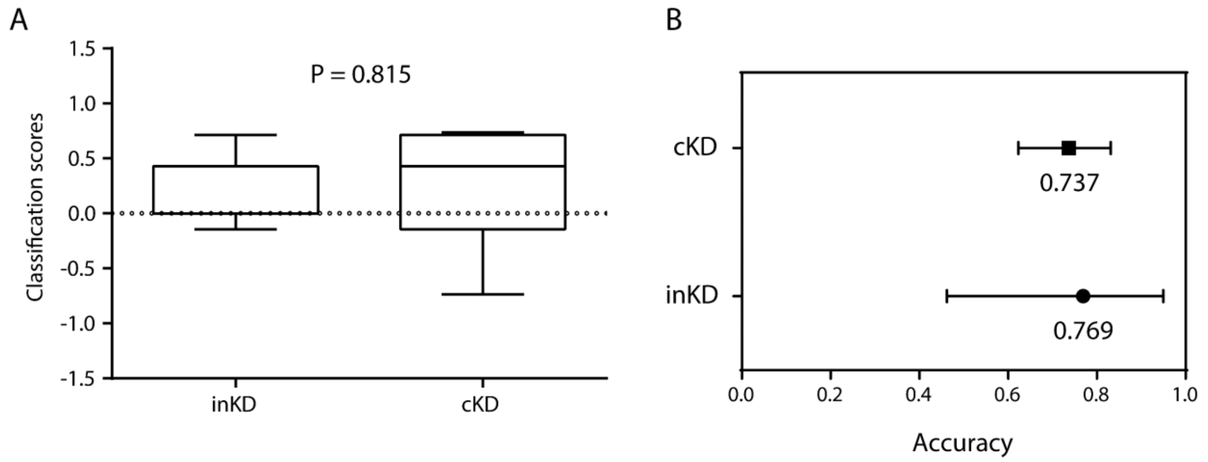

Supplementary Figure S7. The prediction performance of TRGP classifier in the cKD and inKD patients. (A)The classification scores of cKD and KD patients in GSE68004. P value shows the Two-tailed unpaired Student's t-test for the classification scores between cKD and KD. (B)The prediction accuracy of the cKD and KD. Whiskers represent the range of 95% percentile range, and symbols represent the mean value.
