# Supplementary material for: Distinguishing Kawasaki Disease from Febrile Infectious Disease Using Gene Pair Signatures
Source: Biomed Res Int. 2020 Apr 26;2020:6539398. doi: 10.1155/2020/6539398 (PMC7201505; doi:10.1155/2020/6539398)
Supplement: Supplementary 2 — Table 1: the 2522 top scores gene pairs and the 60 top-ranked gene pairs. Supplementary Table 2: the performance of TRGP classifier in different GEO data sets. Supplementary Table 3: the importance of 12 gene pair signatures in the SVM classifier. Supplementary Table 4: the detailed information of each study of the discovery validation. [file 6539398.f2.zip › Supplementary Materials-Table2-4_BMRI_3051554.docx]

Supplementary Table 2. The performance of TRGP classifier in different GEO data sets.

| Data sets | | | Discovery set |  | Validation set-1 | |  | Validation set-2 | | | | | | |
| --- | --- | --- | --- | --- | --- | --- | --- | --- | --- | --- | --- | --- | --- | --- |
| GEO Accession | | | GSE73461 |  | GSE73462 | GSE73463 |  | GSE48498 | GSE16797 | GSE68004 | GSE40396 | GSE38900 | GSE22098 | |
| Accuracy | | | 0.83 |  | 0.863 | 0.959 |  | 1 | 0.941 | 0.6 | 0.767 | 0.828 | 0.282 | |
| Sensitivity | | | 0.936 |  | NA | 0.959 |  | 1 | 0.941 | 0.742 | NA | NA | NA | |
| Specificity | | | 0.774 |  | 0.863 | NA |  | NA | NA | 0.25 | 0.767 | 0.828 | 0.282 | |
| Precision | | | 0.689 |  | 0 | 1 |  | 1 | 1 | 0.71 | 0 | 0 | 0 | |
| Balanced Accuracy | | | 0.855 |  | NA | NA |  | NA | NA | 0.496 | NA | NA | NA | |
|  |  | Confusion Matrix | | | | | | | | | | | |  |
| KD(Predicted)-KD(Actual) | | | 73 |  | 0 | 140 |  | 12 | 16 | 66 | 0 | 0 | 0 | |
| FI(Predicted)-KD(Actual) | | | 5 |  | 0 | 6 |  | 0 | 1 | 23 | 0 | 0 | 0 | |
| KD(Predicted)-FI(Actual) | | | 33 |  | 7 | 0 |  | 0 | 0 | 27 | 10 | 26 | 28 | |
| FI(Predicted)-FI(Actual) | | | 113 |  | 44 | 0 |  | 0 | 0 | 9 | 33 | 125 | 11 | |

Supplementary Table 3. The importance of 12 gene pairs signatures in SVM classifier.

| Gene pairs ID | Gene i EntrezID | Gene j EntrezID | Gene i Symbol | Gene j Symbol | SVM features coefficients |
| --- | --- | --- | --- | --- | --- |
| GenePair46 | 7273 | 79411 | TTN | GLB1L | 2.270 |
| GenePair43 | 84795 | 116832 | PYROXD2 | RPL39L | 1.800 |
| GenePair13 | 22884 | 23077 | WDR37 | MYCBP2 | 2.433 |
| GenePair3 | 10857 | 7384 | PGRMC1 | UQCRC1 | 1.843 |
| GenePair31 | 8848 | 3191 | TSC22D1 | HNRNPL | 1.594 |
| GenePair27 | 8436 | 54460 | CAVIN2 | MRPS21 | -1.920 |
| GenePair30 | 3553 | 1509 | IL1B | CTSD | 1.116 |
| GenePair23 | 2537 | 3429 | IFI6 | IFI27 | 1.758 |
| GenePair45 | 1071 | 152926 | CETP | PPM1K | 1.464 |
| GenePair8 | 402483 | 5905 | LINC01000 | RANGAP1 | -0.056 |
| GenePair54 | 146225 | 994 | CMTM2 | CDC25B | -0.041 |
| GenePair32 | 5165 | 286530 | PDK3 | P2RY8 | -0.233 |

Supplementary Table 4. The detailed information of each study^a^.

|  | | | **Discovery** | | **Validation set-1** | | **Validation set-2** | | | | | | | | | | | | | | |
| --- | --- | --- | --- | --- | --- | --- | --- | --- | --- | --- | --- | --- | --- | --- | --- | --- | --- | --- | --- | --- | --- |
|  | | |  | |  | |  | | | | | | | | | | | | | | |
| Ref. | | | Wright *et al*. | | Wright *et al* | Wright *et al*. | Ogihara *et al*. | Ogata *et al*. | | | Jaggi *et al*. | | | | | Hu *et al.* | Mejias *et al.* | | | | Berry *et al*. |
| Accession | | | GSE73461 | | GSE73462 | GSE73463 | GSE48498 | GSE16797 | | | GSE68004 | | | | | GSE40396 | GSE38900 | | | | GSE22098 |
| Group | | | KD | FI | FI | KD | KD | KD | | | KD | | | DV | DV | DV | DV | | | | DB |
| Subgroup | | |  |  |  |  |  | Group  A | Group  B1 | Group  B2 | Train Set  (Complete KD) | Test Set  (Complete KD) | Validation Set  (Incomplete KD) | Train  Set | Test  Set |  | Train  set | Test  set | Validation  Set A | Validation  Set B |  |
| No. of patients | | | 78 | 326 | 130 | 72 | 8 | 6 | 6 | 5 | 39 | 37 | 13 | 9 | 10 | 43 | 45 | 46 | 16 | 28 | 51 |
| Age,median(IQR)/median±SD, month | | | 27(16-45) | 37(9-116) | 17(5-47) | 34（17-51) | 50(18-84) | 31±24.1 | 44.0±23.9 | 26.0±24.0 | 45.6  (25.2-61.2) | 33.6  (16.8-70.8) | 49.2  (21.6-78.0) | 58.8  (22.8–81.6) | 51.6  (22.8–64.8) | 14.0  (5.5-22) | 2.3  (1.6-6.3) | 1.6  (0.7-2.9) | 6.5  (4.0-12.7) | 2.9  (1.6-4.3) | 83  (33-132) |
| Male sex,No.(%) | | | 43（55.1） | 184（56.4） | 74（56.9） | 45（62.5） | 6（75） | 3（50） | 4（66.7） | 3（60） | 23（59） | 21（57） | 8（62） | 7(77.8) | 3(30.0) | 25(58.1) | 24(53) | 22(48) | 7(44) | 17(61) | 26（50.9） |
| Illnes day at diagnosis,median(IQR)/median±SD, day | | | 5（4-6） | 6（4-9） | 5(3-7) | 5(5-6) | 4(2-6) | 5.0±0.9 | 3.5±1.0 | 4.0±1.1 | NA | NA | NA | NA | NA | NA | NA | NA | NA | NA | NA |
| **Ethnicity , No.(%)** | |  |  | | | | | | | | | | | | | | | | | | |
| Black | | | 3（3.8） | 28(8.6) | 23(19.2) | 2(2.8) | 0 | 0 | 0 | 0 | 9（23） | 9（24） | 1（8） | 1(11.1) | 1(10) | 16(37.2) | 2(4) | 4(9) | NA | 3(11) | 21(41.2) |
| White | | | 20（25.6） | 186(57.1) | 68(56.7) | 20(27.8) | 0 | 0 | 0 | 0 | 22（56） | 16（43） | 9（69） | 4(44.4) | 9(90) | 25(58.1) | 9(20) | 10(22) | NA | 22(79) | 13(25.4) |
| Hispanic | | | 25（32.1） | 20(6.1) | 0 | 14(19.4) | 0 | 0 | 0 | 0 | 3（8） | 2（5） | 1（8） | 0 | 0 | 0 | 31(69) | 29(63) | NA | 2(7) | 17(33.3) |
| Asian and Other^b^ | | | 30(38.5) | 69(21.2) | 29(22.3) | 36(50) | 8(100) | 6(100) | 6(100) | 5(100) | 5（13） | 10（27） | 2（15） | 4(44.4) | 0 | 2(4.7) | 3(7) | 3(6) | NA | 1(3) | 1(2.0) |
| **Loaboratory values, median(IQR)/median±SD** |  |  | | | | | | | | | | | | | | | | | | | |
| White blood cell count, ×103/μl | | | 14.2(10.1-18.3) | 8.0(6.0-12.9) | 11.0(7.7-16.0) | 13.9(11.0-19.0) | 16.8(13.7-21.0) | 14.5±7.2 | 11.0±6.3 | 12.0±3.6 | 13.3(10.5-20.2) | 13.5(10.3-18.3) | 15.4(10.5-19) | NA | NA | 15.8(10.1-19.8) | 10.0(7.7-11.7) | 10.9(8.8-14.9) | 10.0(7.3-12.6) | 12.0(9.2-16.6) | NA |
| Neutrophil count, ×103/μl | | | 9.0(6.6-12.4) | 5.0(3.1-9.4) | 7.0(3.6-13.4) | 10.0(7.3-12.6) | 11.0(9.7-15.3) | 9.5±6.1 | 8.2±5.9 | 9.0±1.7 | NA | NA | NA | NA | NA | 10.1(5.8-12.8) | NA | NA | NA | NA | NA |
| Platelet count,×103/μl | | | 352(303-448) | 254(167-351) | 277(176-352) | 408(324-474) | 433(349-631) | 341±119 | 279±96 | 270±106 | NA | NA | NA | NA | NA | NA | NA | NA | NA | NA | NA |
| CRP mg/L | | | 119(48-192) | 66(23-174) | 62(16-162) | 87(59-173) | 87(51-116) | 93±70 | 94±45 | 62±50 | 137(56-173) | 108(47-154) | 109(57-181) | NA | NA | NA | NA | NA | NA | NA | NA |
| Z-max score | | | NA | NA | NA | NA | 2.6(1.3-15.0) | NA | NA | NA | NA | NA | NA | NA | NA | NA | NA | NA | NA | NA | NA |
| Coronary artery status^c^ % | | |  |  |  |  |  |  |  |  |  |  |  |  |  |  |  |  |  |  |  |
| Normal | | | 45（57.7） | NA | NA | 52（72.2） | NA | NA | NA | NA | NA | NA | NA | NA | NA | NA | NA | NA | NA | NA | NA |
| Dilated | | | 25（32.1） | NA | NA | 15（20.8） | NA | NA | NA | NA | NA | NA | NA | NA | NA | NA | NA | NA | NA | NA | NA |
| Aneurysm | | | 8（10.3） | NA | NA | 5（6.9） | NA | NA | NA | NA | NA | NA | NA | NA | NA | NA | NA | NA | NA | NA | NA |

Abbreviations: KD, Kawasaki Disease; FI, febrile infection; DV, defined viral infection; DB, defined bacterial infection; IQR, interquartile range; SD,standard deviation; CRP, C-reactive protein; NA, not applicable.

^a^ The clinical characteristics and laboratory values in this table were obtained from each study, and not all the patients met the inclusion criteria in the present study(see Materials and Method Section, Data and pre-processing), these data could only show the approximate situation of the Discovery set, Validation set-1 and Validation set-2.

^b^ Asian and Other, including Asian,Indian subcontinent, Far East and Mixed race.

^c^ Normal, Z-max score < 2.5; Dilated, Z-max score ≥ 2.5 & < 5; Aneurysm, Z-max score ≥ 5.
